# Impact of closed-system drug transfer device on exposure of environment and healthcare provider to cyclophosphamide in Japanese hospital

**DOI:** 10.1186/2193-1801-2-273

**Published:** 2013-06-21

**Authors:** Tomohiro Miyake, Takuya Iwamoto, Manabu Tanimura, Masahiro Okuda

**Affiliations:** Department of Pharmacy, Ise Red Cross Hospital, Mie, Japan; Department of Clinical Pharmacy and Biopharmaceutics, Mie University Graduate School of Medicine, Mie, Japan; Department of Pharmacy, Mie University Hospital, Mie, Japan

**Keywords:** Cyclophosphamide, Surface contamination, exposure of healthcare provider, PhaSeal, Japanese guidelines

## Abstract

In spite of current recommended safe handling procedures, the potential for the exposure of healthcare providers to hazardous drugs exists in the workplace. A reliance on biological safety cabinets to provide total protection against the exposure to hazardous drugs is insufficient. Preventing workplace contamination is the best strategy to minimize cytotoxic drug exposure in healthcare providers. This study was conducted to compare surface contamination and personnel exposure to cyclophosphamide before and after the implementation of a closed-system drug transfer device, PhaSeal, under the influence of cleaning according to the Japanese guidelines. Personnel exposure was evaluated by collecting 24 h urine samples from 4 pharmacists. Surface contamination was assessed by the wiping test. Four of 6 wipe samples collected before PhaSeal indicated a detectable level of cyclophosphamide. About 7 months after the initiation of PhaSeal, only one of 6 wipe samples indicated a detectable level of cyclophosphamide. Although all 4 employees who provided urine samples had positive results for the urinary excretion of cyclophosphamide before PhaSeal, these levels returned to minimal levels in 2 pharmacists after PhaSeal. In combination with the biological safety cabinet and cleaning according to the Japanese guidelines, PhaSeal further reduces surface contamination and healthcare providers exposure to cyclophosphamide to almost undetectable levels.

## Background

Many drugs used in the treatment of cancer are considered to be hazardous to healthcare workers. Over the last 20 years, several studies have reported environmental contamination with hazardous drugs in hospital pharmacies (Castiglia et al. 2008; Ensslin et al. 1994; Hedmer et al. 2008; McDevitt et al. 1993; Sessink et al. 1992; Sessink et al. 1995; Sugiura et al. 2011; Vandenbroucke and Robays 2001; Yoshida et al. 2011). In addition, hazardous drugs were inadvertently absorbed, as determined by the presence of parent compounds and/or their metabolites in the urine of health care workers ( Ensslin et al. 1997; Schreiber et al. 2003; Sessink et al. 1992;Sessink et al. 1994; Sessink et al. 1997). Due to the potential health risks of hazardous drugs, the increasing use of these drugs, and continuing environmental contamination, the National Institute for Occupational Safety and Health (NIOSH) published an alert for antineoplastic and other hazardous drugs used in healthcare settings (National Institute for Occupational Safety and Health NIOSH 2004). Based upon recommendations, the American Society of Health-System Pharmacists (ASHP) and the International Society of Oncology Pharmacy Practitioners (ISOPP) have published updated guidelines on the safe-handling of cytotoxic and hazardous drugs (American Society of Health-System Pharmacists guidelines on handling hazardous drugs 2006; International Society of Oncology Pharmacy Practitioners Standards Committee. ISOPP standards of practice 2007).

In Japan, guidelines for handling antineoplastic drugs in hospitals were issued by the Japan Pharmaceutical Association in 1991, which was updated in 1994. Furthermore, these guidelines were revised and published as “Compounding Manuals for Antineoplastic Agents” in 2005 and In Japan, guidelines for handling antineoplastic drugs in hospitals were issued by the Japan Pharmaceutical Association in 1991, which was updated in 1994. Furthermore, these guidelines were revised and published as “Compounding Manuals for Antineoplastic Agents” in 2005 and In Japan, guidelines for handling antineoplastic drugs in hospitals were issued by the Japan Pharmaceutical Association in 1991, which was updated in 1994. Furthermore, these guidelines were revised and published as “Compounding Manuals for Antineoplastic Agents” in 2005 and 2009 (Kitada et al.). The Japanese Society of Hospital Pharmacists (JSHP) academic committee then updated and published the “Guidelines for Compounding Antineoplastic Agents,” referring to the “ALERT” in “Preventing Occupational Exposure to Antineoplastic and Other Hazardous Drugs in Health Care Settings” announced by the NIOSH and guidelines from the ASHP.

Introduction of the biological safety cabinet (BSC) for the preparation of anticancer drugs is limited, with only 35.2% of hospitals using the BSC in Japan, even though guidelines on the preparation of anticancer drugs exist( JSHP. 2012 ). Recently, the advantage of closed-system drug transfer device (CSTD) is recognized to prevent or reduce exposure of healthcare providers from hazardous drugs. The CSTD is a device that mechanically prevents contamination of the environmental substances into a drug solution and the escape of hazardous drug or vapor concentrations outside the system. In addition, only 10.7% of hospitals in Japan currently use the CSTD ( JSHP. 2012 ), although pharmacists in 90% or more of hospitals recognize the usefulness of the CSTD. Reimbursement of technical fees for the use of CSTD to the medical institutions under the medical insurance system were introduced in 2010, and the value was raised in 2012 for the preparation of volatile anticancer drugs, i.e. cyclophosphamide, ifosfamide and bendamustine; therefore, the use of the CSTD in a hospital setting has been increasing in Japanese hospitals. Interestingly, there is no authorized pharmacy technician system in Japan, and, as such, pharmacists are regarded as being primarily in charge of compounding hazardous drugs.

At Yamada Red Cross Hospital (current name: Ise Red Cross Hospital), we developed institutional manuals for compounding anticancer drugs in reference to the above guidelines and began to use BSC Class IIB2 and personal protective equipment (PPE) in the compounding room of the pharmacy department. Each pharmacist in charge of compounding anticancer drugs wear two layers of gloves, a disposable polypropylene gown with long sleeves and closed fronts, a disposable cap and a disposable surgical mask. In addition, the BSC and floor of the compounding room are wiped after compounding according to the Japanese guidelines. However, we revealed that cyclophosphamide (CP) was still detected at the sites of the wipe tests as well as in urine samples from all pharmacists in charge of CP compounding ( Tanimura et al. 2009 ). Several studies have shown nearly complete containment or reduction in surface contamination accompanying preparation and/or administration of hazardous drug ( Connor et al. 2002 ; Vandenbroucke and Robays 2001 ; Wick et al. 2003 ).

We conducted this study to evaluate the effects of the CSTD on surface contamination and exposure of pharmacists in charge of compounding CP in a Japanese hospital setting.

## Materials and methods

Yamada Red Cross Hospital is a community hospital in Mie prefecture Japan with 655 beds for inpatients designated for the treatment of cancer. In the hospital, 7061 patients were treated with anticancer agents in the year 2010. The study design was approved by the Institutional Review Board at Yamada Red Cross Hospital and all subjects provided written.

### Wipe tests

**Figure 1:**
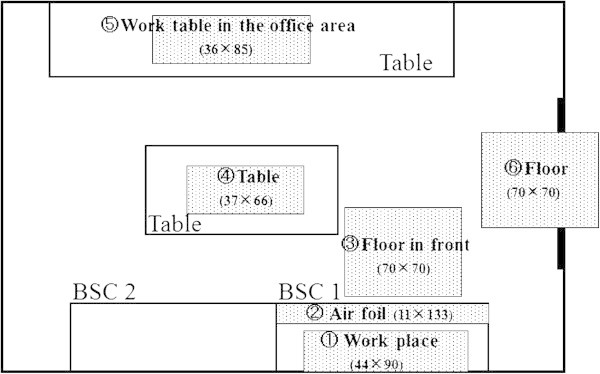
**Sites of the wipe test in the chemotherapy preparation room at the pharmacy.**

A total of 6 sites in the anticancer drugs compounding room of the pharmacy were subjected to wipe tests for CP (Shionogi & Co., Ltd., Osaka, Japan) contamination on September 7, 2007 (first test) using Cyto Wipe Kits (Exposure Control B.V., Al Wijchen, Netherlands). On April 2008, we began to use the CSTD (PhaSeal^®^; Carmel Pharma ab, Goteborg, Sweden), and then the same tests were conducted on March 4, 2009 (second test) eleven months after the implementation of PhaSeal. The same pharmacist collected the wipe samples, and this study was faithfully done according to the wipe manual. The test sites were: 1. work surface of the BSC; 2. airfoil inside the BSC; 3. the floor in front of the BSC; 4. the central work table; 5. the work table with a telephone and personal computer in the office area; and 6. the floor of the entrance into the compounding room (Figure 1). We wiped exactly the same position of surface before and after implementation of PhaSeal by assuring the distance from the edge of the wall and the table. All wipe samples were taken at the end of the routine preparation of hazardous drugs before daily cleaning. We used 0.03M sodium hydroxide, 2% sodium hypochlorite, and 1% sodium thiosulfate to wipe clean the surface of BSC. The floors of the room were cleaned using disposable sheets, which were dampened with ethanol and detergent. The tables were wiped using a disposable nonwoven rayon fabric dampened with ethanol. The floors and tables of the room were wiped every day.

Cyto Wipe Kits were used for all sampling. These kits contained standardized supplies for taking samples, including certified drug-free sampling tissues, dropper bottles containing sampling solution, storage containers, latex gloves, and labels. Each predetermined sampling location was marked with colored tape and measured to determine the sample area. All sample containers were pre-labeled with the collection date and a coded number that identified the study site and sample location.

### Urine test

Four pharmacists (3 males and 1 female) who compounded CP collected their own urine samples over 24 h. It would be collected for 24 h during the period of August 30 through September 11, 2007 based on the schedule of the pharmacist. These pharmacists collected urine samples at every urination from prior to compounding anticancer drugs to the next morning. More than 7 months after the initiation of PhaSeal, urine samples were collected again from the same 4 pharmacists between November 7, 2008 and March 17, 2009.

As stipulated in the instruction manual, we collected samples using Cyto Urine Kits (Exposure Control B.V., Al Wijchen, Netherlands). Using measuring cups, we took urine samples from the test pharmacists. The sampling times and volume of urine were recorded. We placed 5 mL of the urine collected into a plastic container with a screw-on lid. The containers were stored in a freezer at -80 degrees C. The amount of CP prepared by these pharmacists on the day of the urine test was also recorded. All 4 pharmacists did not take any medicines that may have influenced CP metabolism.

### Analysis of samples

Frozen wipe and urine samples were transported with dry ice to Exposure Control for determination of CP level. Samples were analyzed with a Varian Saturn 4D GC-MS/MS ion-trap system with a Varian 8200 auto sampler, controlled by a computer. The detection limits of intact CP were 0.10 ng/mL for the wipe test and 0.01 ng/mL for the urine test.

## Results

### Wipe tests

The results of the wipe tests were shown in Table 1. The contamination per cm^2^ was calculated. Before PhaSeal, CP was detected at 4 test sites in the compounding room in the pharmacy: 1. the work surface; 2. airfoil inside the BSC; 3. the floor in front of the BSC; 6. the floor of the entrance to the compounding room. After the installation of PhaSeal, CP was not detected in any other locations except for the floor of the entrance to the compounding room.

**Table 1:** **Concentration of CP in wipe samples** (**ng**/**cm**^**2**^) **in the preparation on room**

				First test				Second test		
Sample	Description spot	Area (cm^2^)		ng/mL (NaOH)	ng	ng/cm^2^		ng/mL (NaOH)	ng	ng/cm^2^
1	Work place	3900		0.28	45	0.012		nd	-	-
2	Air foil	1463		0.54	86	0.059		nd	-	-
3	Floor in front	4900		0.17	27	0.006		nd	-	-
4	Table	2442		nd	-	-		nd	-	-
5	Office area	3060		nd	-	-		nd	-	-
6	Floor	4900		0.15	24	0.005		0.24	38	0.008

*nd* not detected. The first test was conducted on September 7, 2007. The second test was conducted on March 4, 2009. The contamination per cm^2^ was calculated assuming 100% recovery and wipe efficiency. The detection limit for cyclophosphamide was 0.10 ng/mL NaOH. PhaSeal^®^ was not used for the first test, and was used for the second.

### Urine test

The mean amount of CP compounded by pharmacists on the day of the urine test after the installation of PhaSeal was 11.9% (3945 mg) higher than that before the installation of PhaSeal (3525 mg). Before PhaSeal, 34 urine samples were collected from four pharmacists, and CP was detected in 26 samples. The total amount of CP excreted in each pharmacist was 34.9, 27.0, 56.5, and 71.3 ng/24 h (Table 2), respectively, with mean value 47.4 ng/24 h. After PhaSeal, 31 urine samples were collected from the same four pharmacists, and CP was detected in two urine samples only from two pharmacists. The total amount of CP in these samples was 6.4 and 7.8 ng/24 h, respectively, with mean value 3.6 ng/24 h (Table 2).

**Table 2:** **Total amount of CP in urine samples (ng/24 h) from four pharmacists**

Pharmacist	Amount of CP prepared (mg/day)	Number of samples	Number of positive samples	Detection rate (%)	CP (ng/24 h)
First test (before PhaSeal^®^)					
1	2900	10	7	70.0	34.9
2	2810	9	6	66.7	27.0
3	3530	8	6	75.0	56.5
4	4860	7	7	100.0	71.3
Mean	3525	8.5	6.5	77.9	47.4
Second test (after PhaSeal^®^)					
1	3000	8	0	0.0	nd
2	4600	7	0	0.0	nd
3	3700	8	1	12.5	6.4
4	4480	8	1	12.5	7.8
Mean	3945	7.8	0.5	6.3	3.6

nd: not detected.

The first test was conducted between August 30 and September 11, 2007.

The second test was conducted between November 7, 2008 and March 17, 2009.

The amount of CP prepared was measured on the day of the urine test.

PhaSeal^®^ was not used for the first test, and was used for the second test.

## Discussion

Several steps in the compounding anticancer drugs create conditions that may allow the escape of the drug into the compounding room as well as the work surface of BSC. A common source of contamination is the formation of an aerosol due to over or under pressure inside the drug vial. An aerosol forms when the vial membrane is punctured and the air pressure within the vial quickly equalizes with ambient air pressure. The aerosol settles on work surfaces and other exposed areas in the immediate environment. Working with hazardous drugs in health care settings has demonstrated measurable urine concentrations accompanied within fertility, miscarriage, and birth defects ( Selevan et al. 1985 ; Valanis et al. 1997 ; Valanis et al. 1999 ). Cautionary reports described that lymphocyte DNA damage and chromosome 5 and 7 abnormalities may be induced in healthcare providers handling hazardous drugs ( McDiarmid et al. 2010 ; Sasaki et al. 2008 ; Yoshida et al. 2006 ). Although the BSC may reduce environmental contamination by an aerosol and spills of hazardous drugs, it cannot completely prevent environmental contamination.

The Japanese guideline “Guideline for aseptic handling of injection and antineoplastic drugs” was developed referring to the guidelines of the ASHP and ISOPP ( Nabeshima et al. 2008 ). In addition to the use of the CSTD, cleaning of the BSC is also recommended. We reported that CP was detected in 4 wipe samples with the mean CP concentration being 0.014 ng/cm^2^ before PhaSeal was used; however, CP was detected in only 1 wipe sample at 0.001 ng/cm^2^ after PhaSeal was installed. The mean amount of CP in urine samples was also reduced to 7.1% with PhaSeal relative to that before PhaSeal. There are three features in our study. 1; The training of pharmacists to use PhaSeal. In the present study, the effect of PhaSeal was evaluated more than 7 months after the implementation of PhaSeal. The importance of training for using the CSTD has also been described in the guidelines of the ISOPP (International Society of Oncology Pharmacy Practitioners Standards Committee. ISOPP standards of practice 2007). 2; Method of cleaning the environment. There is an increased risk of inhaling CP when the inside of the BSC being cleaned with ethanol ( Pethran et al. 2003 ). We prevented the inhalation of hazardous drugs by cleaning the compounding room according to the Japanese guideline ( Nabeshima et al. 2008 ). The inside of the BSC was wiped down 4 times with gauze, which were consecutively moistened with 0.03 M NaOH, 2% sodium hypochlorite, 1% sodium thiosulfate, and 80% ethanol ( Mochizuki et al. 2008 ). The floor was also cleaned using disposable sheets every day. 3; Analysis of samples. Our samples were measured by Exposure Control. A few wipe samples were collected in this study, because of the small number of pharmacists and limited budget for the cost of analytical methods. Therefore, our results were shown by the mean value. However our result indicates that exposure to CP is decreased by using PhaSeal and cleaning the BSC and floor according to the guidelines.

In the US, four quantitative studies have been conducted to evaluate the use of PhaSeal in reducing contamination of the workplace with hazardous drugs ( Connor et al. 2002 ; Harrison et al. 2006 ; Nyman et al. 2007 ; Wick et al. 2003 ). Sessink et al. tested PhaSeal in 22 US hospitals and concluded that a marked reduction in environmental contamination could be achieved if the compounding was performed using PhaSeal ( Sessink et al. 2011 ). In Japan, Yoshida reported that CP was detected in all wipe samples, and also indicated that the mean amount of CP in urine samples after the installation of PhaSeal ( Yoshida et al. 2009 ). These reports are comparable with our current study that reduction of healthcare provider exposure to hazardous drugs was achieved by standard handling of the drug with the CSTD in accordance with Japanese guidelines.

Our results show that a reduction in environmental contamination can be achieved if the preparation is performed by using PhaSeal. However, even with the use of the PhaSeal, environmental contamination was still observed. Possible sources contributing to this observation may include remaining contamination from the past and introduction of new contamination via external contamination on the drug vials. Hedmer et al. ( 2005 ) and Connor et al. ( 2005 ) had been published showing surface contamination of antineoplastic drug vials. While our current study show reduction in contamination, it was difficult to evaluate exactly whether PhaSeal completely prevent the exposure of healthcare professionals to CP because significant amount of CP may be carried in from the package surface of CP during compounding.

Up to 25% of CP administered is excreted in the urine as an unchanged form for 24 h, and more than 50% of CP is metabolized to 4-ketocyclophosphamide and carboxyphosphamide via 4-hydroxycyclophosphamide by cytochrome P450 and oxidase in the liver ( Fujita Fujita 1986 ). Tanimura et al. ( 2009 ) reported that 0.24 ng/mL CP was detected at 26 h after compounding CP. It is obvious that detectable levels of CP and its metabolites are still present in the urine of the pharmacists. Since exposure to CP over a long period of time may increase the risk of the accumulation of its metabolites in the body, further studies are needed to clarify the exposure evaluation including the urinary metabolized CP level. Sessink described the risk of developing additional cancer due to systematic exposure to CP in his report ( Sessink et al. 1995 ). By extra polating animal studies to patient data, the calculated chance of developing additional cancer was between 1.4 and 10 additional cancer cases per year per million workers when exposed daily to CP with a urinary excretion value of 180 ng/24 h. When applied to our results, the risk of developing additional cancer due to occupational exposure to CP is shown in Table 3. Our results indicated that the risk of developing additional cancer could be reduced by the combined use of the BSC and CSTD.

**Table 3:** **The risk of developing additional cancer due to occupational exposure to CP**

First test (before PhaSeal^®^)			Second test (after PhaSeal^®^)		
Pharmacist	CP (ng/24 h)	Cancer risk^*^	Pharmacist	CP (ng/24 h)	Cancer risk^*^
1	34.9	0.27 -1.94	1	nd	-
2	27.0	0.21 -1.50	2	nd	-
3	56.5	0.44 - 3.14	3	6.4	0.05-0.36
4	71.3	0.55 -3.96	4	7.8	0.06-0.43

nd: not detected.

The first test was conducted on September 12, 2007.

The second test was conducted on March 18, 2009.

*Cancer risk: extra cancer cases in a million workers each year.

Our results also indicated that the PhaSeal system and cleaning the environment reduced the exposure of employees to CP and its surface contamination in the setting of a Japanese hospital. Therefore, the continuous monitoring of personnel and the environment contamination is necessary to evaluate newly-installed procedures and the long-term effects of its procedures.

## Conclusion

In combination with cleaning according to the Japanese guidelines, PhaSeal further reduces surface contamination and healthcare provider exposure of CP could be achieved at almost undetectable levels.

## Abbreviations

ASHP: American Society of Health-System Pharmacists
BSC: Biological safety cabinet
CP: Cyclophosphamide
CSTD: Closed-system drug transfer device
ISOPP: International Society of Oncology Pharmacy Practitioners
JSHP: Japanese Society of Hospital Pharmacists
NIOSH: National Institute for Occupational Safety and Health
PPE: Personal protective equipment.
